# Direct Cloning of Isogenic Murine DNA in Yeast and Relevance of Isogenicity for Targeting in Embryonic Stem Cells

**DOI:** 10.1371/journal.pone.0074207

**Published:** 2013-09-13

**Authors:** Claes Andréasson, Anna J. Schick, Susanne M. Pfeiffer, Mihail Sarov, Francis Stewart, Wolfgang Wurst, Joel A. Schick

**Affiliations:** 1 Department of Molecular Biosciences, The Wenner-Gren Institute, Stockholm University, Stockholm, Sweden; 2 Physiologisches Institut, Ludwig-Maximilians-Universität, Munich, Germany; 3 Institute of Developmental Genetics, Helmholtz Zentrum Munich, Munich-Neuherberg, Germany; 4 Technische Universität München, Freising-Weihenstephan, Germany; 5 The TransgeneOme Project Group, Max Planck Institute of Molecular Cell Biology and Genetics, Dresden, Germany; 6 Genomics, BioInnovationZentrum, Technische Universität Dresden, Dresden, Germany; IGBMC/ICS, France

## Abstract

Efficient gene targeting in embryonic stem cells requires that modifying DNA sequences are identical to those in the targeted chromosomal locus. Yet, there is a paucity of isogenic genomic clones for human cell lines and PCR amplification cannot be used in many mutation-sensitive applications. Here, we describe a novel method for the direct cloning of genomic DNA into a targeting vector, pRTVIR, using oligonucleotide-directed homologous recombination in yeast. We demonstrate the applicability of the method by constructing functional targeting vectors for mammalian genes *Uhrf1* and *Gfap.* Whereas the isogenic targeting of the gene *Uhrf1* showed a substantial increase in targeting efficiency compared to non-isogenic DNA in mouse E14 cells, E14-derived DNA performed better than the isogenic DNA in JM8 cells for both *Uhrf1* and *Gfap*. Analysis of 70 C57BL/6-derived targeting vectors electroporated in JM8 and E14 cell lines in parallel showed a clear dependence on isogenicity for targeting, but for three genes isogenic DNA was found to be inhibitory. In summary, this study provides a straightforward methodological approach for the direct generation of isogenic gene targeting vectors.

## Introduction

Since its first demonstration [1], gene targeting in embryonic stem (ES) cells has evolved to be a routine technique to modulate gene function in vivo. The sequential steps of genomic DNA isolation, vector construction, homologous recombination in ES cells and chimaera production have seen increases in efficiency that have accelerated the number of new mutants each year such that more than 16,000 targeted mutations in mouse ES cells have already been made [2], [3]. Indeed, the first encyclopedia of mutations in the mouse is near to completion, largely due to productivity gains in serial recombination techniques [4]. Although knockout and conditional ablation of genes are the first steps in understanding gene function in vivo, allelic variants such as disease-mimicking point mutations or whole gene replacement (e.g., with human disease variants) are quickly emerging as necessary tools to phenocopy human genetic disease in model organisms. With regard to species relevance or for clinical applications, these mutations are increasingly created in human ES or iPS cells [5]–[10], a process that is still somewhat tedious due to its use in niche applications [11]. Nonetheless, paired with new technologies to increase homologous recombination such as zinc-finger nucleases, TALens, and CRISPR [12]–[15], targeting vectors are employed widely for genome modification.

Genomic DNA cloning is a key step in targeting vector construction. Inclusion of homologous isogenic targeting sequences is common practice to avoid low targeting frequencies caused by single nucleotide or larger polymorphisms such as insertions, deletions or inversions [16]–[18]. Yet, standard methods employed for the modification of genomic-derived DNA sequences are often not optimal for the construction of isogenic targeting constructs. For example, bacteriophage lambda-red based homologous recombination in bacteria has simplified genomic DNA modification [19], but has the prerequisite of a library of mapped BACs or plasmid vectors as a source of genomic DNA. The library requirement hinders its application in, for instance, targeting campaigns of unique human iPS cell lines or in patient-specific gene-therapy, although substantial improvements have been made here as well [20]. Moreover, the lambda-red methodology requires sequential steps in targeting vector construction. Alternative methods generally employ multiple-step PCR, which is challenging for longer sequences from genomic templates and introduces undesirable mutations at higher frequencies than traditional cloning.

Substantial progress has been made with simultaneous multi-fragment cloning in the yeast *Saccharomyces cerevisiae*, in which short homologous sequences are joined by the DNA double-strand break repair machinery [21]–[24]. In particular, larger homologous regions [25] or overlapping oligomers [26] have been successfully used to retrieve genomic DNA from BACs for the introduction into targeting constructs carried on yeast shuttle vectors. Importantly, yeast readily recombines multiple fragments with a restricted plasmid vector that can replicate and be selected for with positive or negative selection, eliminating sequential steps in targeting vector construction and improving throughput.

In this work we sought to extend yeast homologous recombination cloning methodology to directly retrieve DNA fragments from host cellular genomic DNA using oligonucleotides and without the use of intermediate library preparation or PCR steps. We have constructed a novel yeast vector for Retrieval of Targeting sequences with Verbatim Isogenic Regions (pRTVIR, or “pRetriever”) that efficiently retrieves genomic sequences when co-transformed with isolated chromosomal DNA from embryonic stem cells. In a series of experiments involving more than 70 targeting constructs, we find a significant association between correct targeting and isogenic DNA. Therefore, direct DNA cloning from purified genomic DNA in yeast should prove to be a valuable method for sensitive targeting applications.

## Results

### A Yeast Plasmid for Retrieval of Targeting Sequences (pRTVIR) Facilitates the Construction of Targeting Vectors for Embryonic Stem Cells

Cloning overlapping DNA fragments in yeast has been established for rapid cloning of mammalian targeting vectors [23]. This methodology relies on the homologous recombination between mammalian DNA sequences and a shuttle plasmid vector that can replicate and be selected for in both yeast and *Escherichia coli*. We set out to increase the frequency of successful recombination by attempting to minimize the background resulting from unwanted recombination and ligation events. First, we designed a vector for recombination cloning without any sequence homology to the yeast genome. Briefly, an heterologous auxotrophic selection marker *CaURA3* (orotidine-5-phosphate decarboxylase) [27] from *Candida albicans* driven by the *TEF* promoter from *Ashbya gossypii* was combined with replication sequences derived from the yeast episomal 2 micrometer plasmid. *E. coli* replication sequences and a bacterial ampicillin selection marker were introduced in the plasmid resulting in the 4.5 kb plasmid pCA771. When pCA771 is used to transform a *ura3* yeast strain devoid of its endogenous 2 micrometer plasmid ([Cir^0^]) to Ura^+^, no sequence homology exists between this vector and the host DNA, thus effectively eliminating background arising from homologous recombination.

**Figure 1:**
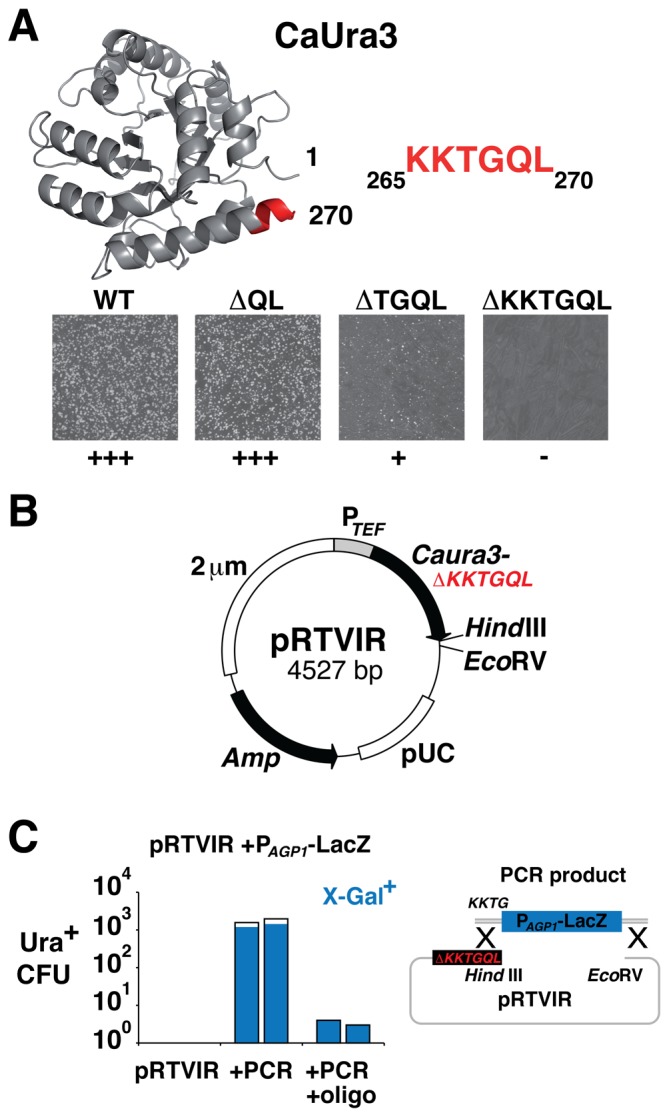
Construction of a plasmid with positive selection for successful recombination (pRTVIR). (**A**) The sequence of *Candida albicans* Ura3 (CaUra3) modeled onto the crystal structure of *Saccharomyces cerevisae* orotidine-5-phosphate decarboxylase [28] using the the Phyre2 server [44]. The very C-terminal 6 residues of CaUra3 are part of a structurally important α-helix (marked in red). *CaURA3* harboring yeast vector pCA771 (WT) was modified by site-directed mutagenesis to remove, 2 (ΔQL), 4 (ΔTGQL) or 6 (ΔKKTGL) of the codons that precede the termination codon of *CaURA3.* Functionality (+++, + or −) of the resulting *CaURA3* markers were assessed by transformation of *ura3*Δ yeast strain CAY1179 followed by plating on media selective for Ura^+^. (**B**) A plasmid map of pRTVIR that contains standard *E. coli* replication (pUC) and antibiotic selection sequences (*Amp*) and replicates in yeast by means of a 2 micrometer plasmid sequence (2 µm). The *Caura3ΔKKTGQL* marker is nonfunctional but can be restored to function by homologous recombination that adds back KKTG codons. *Hind*III and *Eco*RV sites facilitate restriction of pRTVIR that enhances homologous recombination. (**C**). *Hind*III and *Eco*RV restricted pRTVIR was used to co-transform CAY1179 to Ura^+^ in duplicates together with either a **P** *_AGP1_-lacZ* PCR product flanked by homology to the vector (Graphic) or with a similar PCR product without vector homology but in the presence of bridging single-stranded oligonucleotides. Ura^+^ colony forming units (CFU) for each condition are presented. Following plasmid rescue from the yeast transformants, the fractions of correct LacZ-carrying recombinants were quantified by X-Gal blue/white-screening (marked in blue).

Second, we wanted to eliminate background arising from *in vivo* ligation, which in yeast occurs with high frequency [21] by making the functionality of the *CaURA3* selection marker in pCA771 conditionally dependent on a successful recombination event. Briefly, we made small 3′ truncations of the *CaURA3* gene of our pCA771 to define a minimal deletion that abolished function. Deletion of the last two codons of *CaURA3* (ΔQL) did not affect the capacity to transform *ura3* yeast cells to Ura^+^, while a four-codon deletion (ΔTGQL) decreased colony formation frequency and a six-codon deletion (ΔKKTGQL) completely abolished transformation to Ura^+^ (Fig. 1A). Inspection of a structure model of CaUra3 based on the structure of *S. cerevisiae* Ura3 [28] revealed that the deletions had truncated an alpha-helix that is important for the structural integrity of the orotidine-5-phosphate decarboxylase enzyme. We named the new vector pRTVIR, plasmid for Retrieval of Targeting sequences with Verbatim Isogenic Regions (Fig. 1B).

Next, we tested if reintroduction of the four codons KKTG in pRTVIR by homologous recombination restored the activity of *CaURA3* thereby facilitating selection of correct clones as Ura^+^ transformants. The vector was linearized at unique *Hind*III and *EcoR*V restriction sites introduced right at the 3′ end of the *CaURA3*Δ*KKTGQL* coding sequence and was together with a 3.8 kb PCR product encoding a *LacZ* reporter (**P**_AGP1_-*LacZ*) [29] used to cotransform [Cir^0^] *ura3* yeast to Ura^+^. The PCR primers for **P**_AGP1_-*LacZ* had been designed so that they introduced the required KKTG codons together with 30 bp and 35 bp of homology to each of the free ends of the vector, respectively. While control transformations in duplicates using only *Hind*III and *Eco*RV restricted pRTVIR gave no Ura^+^ colonies, inclusion of the **P**_AGP1_-*LacZ* PCR in the transformation gave encouraging 1570 and 1970 Ura^+^ colonies, respectively (Fig. 1C). We pooled the yeast transformants, rescued the plasmids (see Methods) and quantified the number of **P**_AGP1_-*LacZ* carrying recombinants by using X-gal blue/white-screening of the ampicillin resistant *E. coli* colonies. 76% and 73%, respectively, of the transformants were blue and therefore carried a functional *LacZ* gene. In parallel experiments, we employed bridging single- stranded oligonucleotides instead of flanking homology regions to direct the homologous recombination. Briefly, each oligonucleotide was synthesized with 35 bp homology to both one end of the vector and the corresponding end of the **P**_AGP1_-*LacZ* PCR product. Although the yeast transformation efficiency was low in this particular setup due to the use of single and not double stranded oligonucleotides, plasmid rescue and X-gal blue/white-screening revealed 100% efficiency for obtaining correct recombinant plasmids (Fig. 1C).

### Multiple Fragment Targeting Vector Assembly using pRTVIR

**Figure 2:**
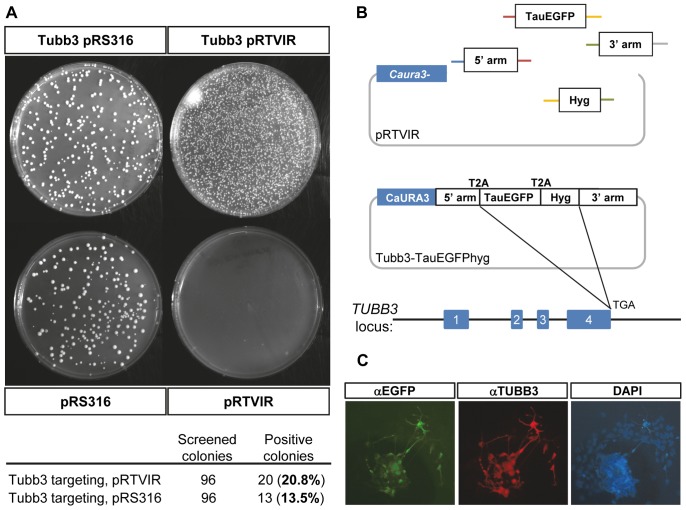
pRTVIR improves multi-fragment recombination and enables gene targeting in mouse ES cells. (**A**). Direct comparison of cloning efficiency following single step multi-fragment recombination of the Tubb3-TauEGFPhyg targeting vector. Single tube transformation of four overlapping PCR fragments (B, top) together with linearized cloning vectors pRS316 or pRTVIR were transformed into competent yeast and transformants plated on solid SC-ura medium plates. Background colony formation was substantially reduced and the frequency of correctly recombined vectors was increased for pRTVIR containing vectors. (**B**). Following multi-fragment recombination containing heterologous 40-bp double-stranded overlapping oligonucleotides ends (colored lines) with pRTVIR, targeting to the penultimate codon of Tubb3 was demonstrated in mouse E14 ES cells. (**C**). In vitro differentiation to neurons shows overlap of EGFP expression corresponding to an antibody directed against Tubb3 in neurons, demonstrating successful targeting following multi-fragment recombination with pRTVIR.

To directly test if pRTVIR functions in homologous recombination of multiple overlapping DNA fragments, a five-fragment mammalian targeting vector for targeting a Tau-EGFP and hygromycin fusion (with adjoining T2A sequences; [30]) to Tubb3 was designed and products were amplified by PCR [31]. We also included a direct comparison with a standard yeast *URA3* vector that contains extensive homology to the yeast genome, pRS316 [32]. In striking contrast to the zero background of pRTVIR upon single-tube transformation, pRS316 gave extensive background of yeast transformants and only modest increase in the number of transformants when inserts were added. Colonies containing correctly recombined fragments were identified by junction PCR and verified by restriction digest. The frequency of correct clones was 20/96 (20.8%) for pRTVIR and 13/96 (13.5%) for pRS316 (Fig. 2). Apparently, plasmid rescue in *E. coli* results in selection of functional plasmids and thereby of correct clones, compensating to some extent for the poor performance of pRS316 during yeast recombination. The recombined pRTVIR-Tubb3-TauEGFPhyg vector was then electroporated into embryonic stem (ES) cells and cells were selected in G418-containing medium. Positively targeted clones (targeting frequency: 4/96) were identified by long-range PCR and analyzed by immunofluorescence (Fig. 2B, S1). As expected, the C-terminal fusion to TauEGFP is localized exclusively in neuronal processes derived from in vitro differentiated ES cells (Fig. 2C), demonstrating that ES targeting vector construction is possible and sequence fidelity is preserved using pRTVIR.

### Cloning of Sequences from BACs and Genomic DNA into pRTVIR

**Figure 3:**
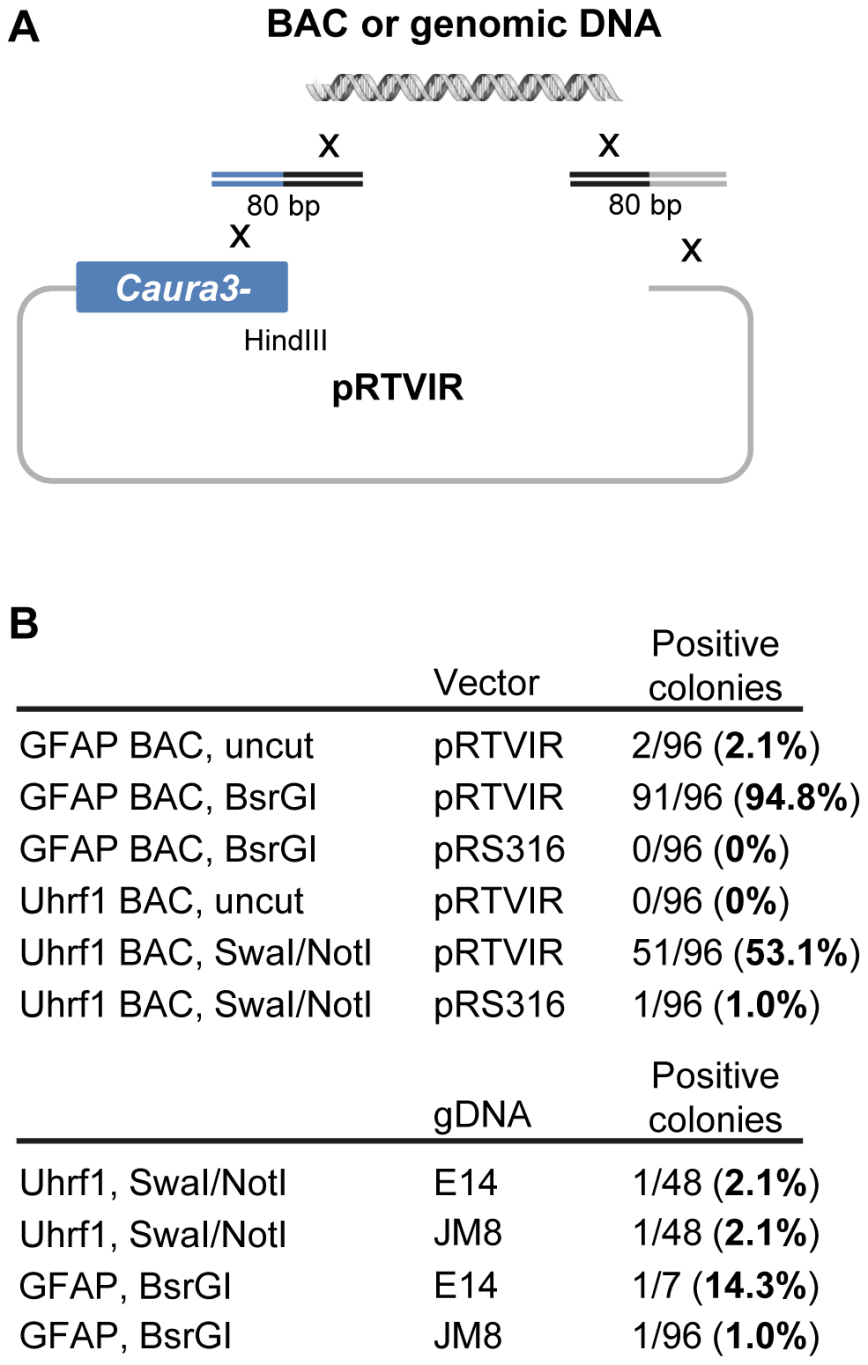
Direct genomic retrieval from BAC and purified genomic DNA into pRTVIR. (**A**). Genomic DNA can be cloned efficiently into pRTVIR in a single step. Freshly purified BAC or genomic was mixed with bridging oligonucleotides containing overlap to the *Caura3-* selection marker and the genomic DNA of interest along with linear pRTVIR. (**B**). Following high efficiency transformation into yeast in triplicate, the frequency of retrieval of 9 kb of unmodified genomic DNA (genomic DNA) from *Gfap* and *Uhrf1* BAC DNA into pRTVIR was slightly increased compared to pRS316. However, introduction of restriction cleavage sites (*Bsr*GI; or *Swa*I, *Not*I) immediately outside the respective genomic region facilitated correct cloning for pRTVIR, but not for pRS316. Due to the improved cloning efficiency of pRTVIR, retrieval of the same region from 100 µg of freshly purified and digested genomic DNA by phenol:chloroform extraction was attempted, giving correctly recombined vectors for both E14- and JM8-derived genomic DNA.

Sensitive genomic applications preclude the use of PCR in mammalian targeting arm amplification due to the frequency of spontaneous mutations. We therefore chose to analyze if pRTVIR would be able to retrieve flanking genomic DNA for a targeting vector directly from a murine bacterial artificial chromosome (BAC). Bridging oligonucleotides containing overlap to the vector and genomic sequence of interest have previously been employed with success [26]. We used a modified protocol to transform a double-stranded primer containing a 40 bp overlap with genomic DNA together with linearized pRTVIR and 40 bp to the corresponding BAC for either *Gfap*, encoding an intermediate filament in astroglia, or *Uhrf1*, encoding a cell cycle regulated E3 ubiquitin ligase (Fig. 3A). Initial results performed in triplicate showed poor retrieval of the ∼9 kb fragments, 2/96 (2.1%) for *Gfap* and 0/96 for *Uhrf1*, but when restriction of the BAC outside the region of interest was introduced, the frequency increased dramatically to 91/96 (94.8%) and 51/96 (53.1%), respectively (Fig. 3B). We also compared the efficiency of linear retrieval in the standard yeast vector pRS316, which gave 0/96 and 1/96 (1.0%), respectively, compared to correctly cloned fragments. This shows that the pRTVIR vector combined with linearization is an efficient tool for retrieval of genomic DNA fragments from linearized BAC DNA.

Genomic DNA isolated directly from cells for targeting experiments has the advantage of absolute isogenicity. Due to the substantial increase in efficiency of genomic DNA retrieval from a BAC over traditional cloning approaches, we attempted to retrieve targeting vector homology directly from genomic DNA derived from embryonic stem cell lines isolated from genetically distinct mouse strains. In experiments conducted in triplicate, following co-transfection of 100 µg of highly purified phenol:chloroform extracted and digested genomic DNA from ES cells together with pRTVIR and overlapping 80-mer double-stranded oligonucleotides corresponding to end sequences at flanking genomic *Swa*I and *Not*I restriction sites, it was possible to retrieve 8.4 kb of *Uhrf1* genomic DNA directly from E14TG2a (129/Ola) and JM8 (C57BL/6) linearized genomic DNA, albeit at a modest frequency of 2.1% (Fig. 3). An additional retrieval gave 14.3% and 1.0% successful clones when isolating 8.4 kb of E14 and JM8 genomic *Gfap* DNA, respectively, digested with flanking *Bsr*GI restriction sites. This demonstrates that selection with the pRTVIR is sufficient to retrieve restricted genomic DNA directly from purified cellular DNA without further amplification steps.

DNA engineering using homologous recombination in yeast does not rely on restriction sites for fusing the fragments and therefore allows for seamless fusion of DNA. To introduce an in-frame fusion of EGFP to *Uhrf1* before the ultimate stop codon, the 8.4 kb *Uhrf1* and *Gfap* homology vectors were introduced into yeast together with a PCR amplified EGFP-SV40 promoter-Neomycin cassette containing 40 bp of homology at the ends. Following transformation and selection on kanamycin, several of the clones screened for *Gfap* and *Uhrf1* correctly contained the cassette (Fig. S2) and were used for targeting experiments described below.

### Targeting with Isogenic and Non-isogenic Yeast-derived Vectors

**Figure 4:**
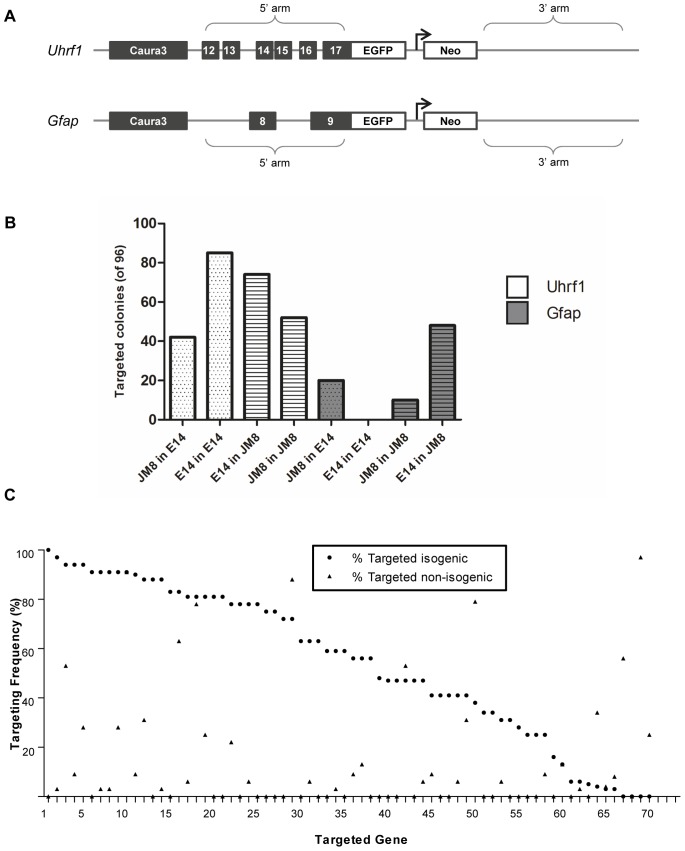
Targeting with isogenic and non-isogenic yeast-derived vectors. (**A**). Yeast-derived targeting vectors for *Uhrf1* and *Gfap* genes are shown. Each vector contains homologous flanking genomic DNA derived directly from ES cell lines and a T2A-EGFP-promoter-Neo cassette cloned in-frame at the penultimate codon with the exon number shown. (**B**). To directly test if yeast-derived isogenic targeting vectors correlate with an increase in targeting frequency, targeting vectors for *Gfap* and *Uhrf1* were electroporated into ES cells. For each gene, matched vectors and cell line (JM8 in JM8; E14 in E14) or reciprocal unmatched (JM8 in E14; E14 in JM8) electroporations were performed individually in parallel and triplicate. 32 colonies were picked from each plate and genotyped by long-range PCR, the number of correctly targeted colonies is shown. Whereas for *Uhrf1*, E14-derived DNA functioned better than JM8 in isogenic (E14 in E14) as well as unmatched (E14 in JM8) cells, isogenic targeting vectors for *Gfap* containing E14 DNA gave no correct colonies (E14 in E14) but performed well in unmatched cells (E14 in JM8). In all cases, E14 DNA performed best except *Gfap* targeting E14 in E14. (**C**). To further examine if isogenic DNA may be less efficient than unmatched DNA in certain cases, an independent targeting campaign of 70 genes was carried out in E14 and JM8 ES cells with tagging EGFP-promoter-neomycin vectors containing C57BL/6 targeting arms. The isogenic targeting frequency in JM8 (% of correctly targeted clones; •) was compared to non-isogenic targeting frequency in E14 cells (▴). A two-tailed paired t-test showed a strong preference for targeting with the isogenic construct (*p*<0.0001), while nine genes showed a preference for unmatched DNA. Gene order is as follows: 1, Lin28; 2, Stag3; 3, Dmap1; 4, Smc4; 5, Thoc4; 6, Auts2; 7, Cnot1; 8, Smc1b; 9, Smc1a; 10, Ube1x; 11, Ssrp1; 12, Ptbp1; 13, Smad2; 14, Thoc2; 15, March7; 16, Ruvbl1; 17, Cdc73; 18, Rad21; 19, Sorbs1; 20, Trim28; 21, Trrap; 22, Apc; 23, Rtf1; 24, Thoc3; 25, Zfp42; 26, Stat3; 27, Wdr61; 28, Esrrb; 29, Niban; 30, Dppa4; 31, Enth; 32, Ep400; 33, Cbx3; 34, Ddx18; 35, Tcfcp2l1; 36, Hipk2; 37, Klf5; 38, Svil; 39, Sf1; 40, Cebpa; 41, Ddx47; 42, Erk2; 43, Smad3; 44, Sox3; 45, Ctr9; 46, Pten; 47, Tbx3; 48, Tcf7l1; 49, U2af65; 50, Mcm2; 51, Tcfap2c; 52, Yeats4; 53, Npas3; 54, Ruvbl2; 55, Shfdg1; 56, Klf4; 57, Ppp4c; 58, Snciap; 59, Snrpf; 60, Nrob1; 61, Olig2; 62, Wdr5; 63, Mbd3; 64, Atxn1; 65, Cnot2; 66, Htt; 67, T (Brachyury); 68, Dab2; 69, Meg3; 70, Tcl1.

Isogenic matching of targeting vector to target cell line is reported to be conducive for robust targeting [16]–[18]. To confirm this for yeast-derived vectors, we conducted a targeting experiment with vectors containing genomic DNA retrieved from either JM8 or E14 ES cell lines for the *Uhrf1* and *Gfap* genes (Fig. 4A). Vectors were prepared in parallel and electroporated in triplicate in JM8 and E14 cell lines and 32 colonies were picked per 10 cm plate (96 total) and analyzed for targeting frequency by long range PCR as well as GFP expression in ES cells and under neural differentiation conditions (Figs. 4B, S2, S3). Surprisingly, isogenicity was not always favored, as the vector derived from the E14 cell line performed better for both genes in JM8 cells than the JM8 isogenic vector (74 versus 52 for *Uhrf1*, 48 versus 10 for *Gfap*). In the reciprocal experiment, the JM8 vector performed as expected better in the JM8 cell line for *Uhrf1* (52 versus 42) but not for *Gfap* (48 versus 20). Perhaps most surprising, the *Uhrf1* E14 vector did not yield positive colonies in E14 cells but targeted 50% of clones correctly in JM8 cells, suggesting that there is an incompatibility when targeting with the isogenic sequence.

Sequence analysis of *Uhrf1* cloned yeast sequences did not reveal changes other than sporadic nucleotide polymorphisms differing from the reference genome (GRCm38) that may affect targeting frequency. However, *Gfap* E14-derived DNA showed substantial polymorphisms in a 245-bp region in a heavily-repeated and alternatively spliced 3′ UTR in exon 8a (Fig. S4). Yeast-cloned JM8 DNA for *Gfap* also showed variation over a neighboring 214-bp region. It is possible that these regions are involved in transcript stability that may lead to a preference of the non-isogenic vector when performing targeting experiments.

Given the disparity between published reports and our targeting outcome, we chose to examine if isogenic DNA improved targeting efficiency under defined circumstances for a larger set of genes. We therefore carried out a targeting campaign of 70 different genes from the DiGtoP resource [33] in E14 and JM8 cells in parallel using recombineering-derived tagging vectors from C57BL/6 genomic DNA. In each vector, the targeting sequences contain an EGFP-promoter-neomycin resistance cassette and are directed to the penultimate codon of each gene to generate an EGFP-fusion to the endogenous gene. By picking 32 colonies from each targeting plate for analysis, a consensus targeting frequency could be applied to a given vector (Fig. 4C). As expected, 66 of 70 vectors gave positive clones for isogenically matched C57BL/6 DNA in JM8 cells, whereas only 38 of 70 the same vectors gave positives in E14 cells. A significant preference for isogenicity was seen in the percentage of successful clones for each gene (*p*<0.0001, two-tailed t-test), as the mean targeting frequency seen in JM8 is 54.5% (±3.69) versus 14.6% (±2.99) in E14 cells. One gene, Ube1x, gave exactly the same number of positives (91%) in both cell lines. Nevertheless, there were several significant exceptions to the isogenicity rule: nine genes (Niban, Erk2, Mcm2, Ataxin1, Cnot2, Htt, Brachyury, Meg3, Tcl1) showed a preference for targeting in E14 with non-isogenic DNA. Surprisingly, these differences are most dramatic for three of the four JM8 untargeted genes: Brachyury (56%), Meg3 (97%) and Tcl1 (25%), suggesting that isogenic sequences may be a hindrance to successful targeting in some instances.

## Discussion

In this study we have developed the novel yeast cloning vector pRTVIR that simplifies the cloning of DNA from single or multiple PCR products, BACs and even facilitates cloning directly from complex genomic DNA preparations. Homologous recombination cloning based on pRTVIR can rely on sequence homology in the flanking ends of PCR products or on oligonucleotides that provides bridging sequence homology for recombination. The methodology outlined in this study can readily be implemented for example when cloning genomic DNA for sequence sensitive applications such as ES gene targeting.

In the broader perspective, pRTVIR is a general-purpose vector for cloning by yeast homologous recombination and is by no means restricted to mammalian genomic DNA sequences. A few specific points regarding the use and design of pRTVIR are noteworthy. First, homologous recombination cloning is not readily compatible with DNA sequences that contain extensive or long repeats. Direct repeats might cause duplications and deletions between these regions and indirect repeats might cause inversions. Therefore, care should be taken when working with such repetitive regions; DNA sequencing and propagation in recombination deficient *E. coli* hosts are imperative. This said, we have actually successfully employed yeast recombination cloning for repetitive regions such as polyglutamine expansions (data not shown). Second, pRTVIR relies on DNA replication sequences from the endogenous 2 micrometer plasmid. This means that by no circumstances should [Cir^+^] yeast strains be used for recombination since this would risk the low background feature of the vector. In [Cir^0^] hosts pRTVIR replicates with largely varying copy numbers, much like the behavior of standard laboratory plasmids for *E. coli* and the vector is therefore not suitable for experiments in yeast cell biology.

Our driving force that led us to develop the pRTVIR technology stems from an interest in ES cell gene targeting [3]. Working with isogenic targeting vectors have long been the gold standard in designing gene-targeting campaigns and has become increasingly important in the functional study of DNA polymorphisms, often linked to disease. Indeed, pRTVIR can readily be implemented in the construction of isogenic targeting constructs, even directly from genomic DNA. Also in our hands we find superior targeting frequencies when employing isogenic vectors, but for individual genes there are exceptions to this rule. We cannot at this point offer an explanation why these specific combinations of DNA donor:recipient score better than isogenic DNA. The vectors we used in our study do not eliminate gene function and would not be expected to cause haploinsufficiency or changes in expression level that may preclude correct targeting. It has been reported that even a 2% strain difference between targeting constructs can lead to a >25 fold in targeting success [34], yet polymorphisms are not expected to act in favor of the non-isogenic construct. Targeting the 3′ UTRs of genes may also lead to a bias, as they are known to be more highly polymorphic than coding regions. Their role in mRNA structure and stability may also reflect a unique DNA structure that should be taken into consideration for its recombinogenic potential. Additionally, recombination hotspots, potential for random integration, repetitive regions and chromatin structure as well as other factors such as imprinting or copy number sensitivity may play a role in the correct integration of targeting construct DNA. In the absence of information defining these features, isogenicity is still the gold-standard for gene targeting campaigns.

With this study we aimed to establish a proof-of-principle for direct retrieval for genomic DNA into a vector system. Although optimization is clearly necessary for large-scale application, this method can be a valuable tool for targeting experiments where absolute genomic integrity is necessary.

## Methods

### Construction of Plasmid Vectors pCA771 and pRTVIR

Parental plasmid pCA771 was constructed by homologous recombination in yeast (Ura^+^) between a 3.4 kb PCR product (primers A and B (Table S1)) encompassing 2 micrometer sequences, ampicillin marker and *E. coli* origin of replication from template pRS423 [35] and a 1.2 kb PCR product encompassing **P**_TEF_-*CaURA3* from template pUG60 [36] (primers C and D). CaURA3 deletion variants of pCA771, including pRTVIR, was constructed by site-directed mutagenesis using oligonucleotides (E, D and F). pRTVIR and complete sequences of this plasmid are available from Addgene (Addgene ID 46365).

### Yeast Transformation and Plasmid Rescue

Yeast strain CAY1179 was used for all experiments and is a spontaneous Ade^-^ derivative of [Cir^0^] strain yAF7 [37] with the genotype (*MAT* **a** ade2-1 *can1-100 his3-11,15 leu2-3,112* trp1-1 *ura3-1*). Yeast was transformed by an adapted protocol described by [38]. Briefly, 10 ml CAY1179 was grown over night in 2×YPD medium at 30°C. In the morning the strain was diluted in fresh 2×YPD medium (3 ml per transformation) and grown at 30°C from starting OD600 = 0.1. After ∼5 h harvest, the cultures had reached OD_600_ = 1.0 and 3 ml of cell culture for each transformation was harvested by centrifugation (1.5 ml Eppendorf tubes) and washed by resuspension and centrifugation in 100 mM LiOAc (1 ml per transformation). 50 µl of carrier DNA (2 mg/ml heat-denatured salmon testis DNA, Sigma D1626 in 100 mM LiOAc) was added together with vector digest and source DNA to the cell pellet. The suspension was briefly mixed by centrifugation followed by the addition of 300 µl 39% PEG 3350 100 mM LiOAc and intense vortexing for 15 s. After heat shock in a water bath at 42°C for 40 min cells were pelleted by centrifugation, resuspended in 150 µl Milli-Q water and plated on solid SC-ura medium. Colonies were observed after 2 days of incubation at 30°C.

For plasmid rescue, single or pooled yeast colonies were picked and standard silica miniprep kits (including Qiaprep, Qiagen) were after lyticase digestion employed on a cell pellet corresponding to ∼20 µl. Lyticase digestion to weaken the yeast cell wall prior DNA extraction was performed by adding 3 µl Zymolyase (ZymoResearch) to the cells resuspended in 200 µl 10 mM Tris-EDTA buffer (or similar cell suspension buffer belonging to the miniprep kit) and incubating for one hour. Tested commercial preparation of lyticase (including Zymolyase) contains a plasmid that makes *E. coli* ampicillin resistant, which necessitate its removal by DNAse I treatment. The plasmid is present in minute amounts but can be detected by transforming *E. coli* with the lyticase solution and selecting for ampicillin resistance. Following lysis, silica column binding, washing and elution, DNA was electroporated into *E. coli* cloning strain DH10B [39].

### P*_AGP1_-LacZ* Recombination Experiments

For experiments involving PCR products carrying flanking homology regions a 3.8 kb **P** *_AGP1_-lacZ* product was PCR amplified with Phusion DNA polymerase from YCpAGP1-lacZ [29] using primers TATAGAGATGCTGGTTGGAATGCTTATTTGAAAAAGACTGGCTAACCTTATTTCCTCATTTCCTCG and TGTGAGCGGATAACAATTTCACACAGGAAACAGCTGTTATTTTTGACACCAGACCAA. For oligonucleotide directed recombination experiments, **P** *_AGP1_-lacZ* without flanking vector homology was amplified using primers CCTTATTTCCTCATTTCCTCG and TTATTTTTGACACCAGACCAAC. For transformation 250 ng of *Hind*III and *Eco*RV restricted pRTVIR was mixed with 5 µl PCR crude product or PCR product and 5 pmol of recombinogenic oligonucleotides (TATAGAGATGCTGGTTGGAATGCTTATTTGAAAAAGACTGGCTAACCTTATTTCCTCATTTCCTCGGCGGCTAAAAAGGG; TGTGAGCGGATAACAATTTCACACAGGAAACAGCTGTTATTTTTGACACCAGACCAACTGGTAATGGTAGCG). Following transformation, cells were plated on selective SC-ura medium and counted. The yeast transformants were pooled and plasmids were rescued to *E. coli* and scored for LacZ by blue/white-screening on LB-medium selective for ampicillin and supplemented with 25 mg/L X-gal (5-bromo-4-chloro-indolyl-β-D-galactopyranoside; Sigma).

### Statistical Analysis

Statistical analysis was done with Prism 5 (GraphPad Software). All values are given as standard error of mean (s.e.m.) unless stated otherwise.

### Generation of Targeting Constructs

#### Tubb3-TauEGFP targeting construct

PCR amplification was used to generate fragments corresponding to the 9 kb flanking genomic DNA in 5′ and 3′ to the stop codon as well as TauEGFP (pTauEGFP [40]), hygromycin (pSectag) and PGK-neomycin (EUCOMM resource), gel-purified and combined with 1 µg pRS316 linearized with *Sac*I/*Kpn*I (New England Biolabs) or pRTVIR (*Hind*III/*Eco*RV; New England Biolabs) and transformed into yeast as described above. T2A sequences to generate separate polypeptides [41] were introduced at ends with heterologous sequences to avoid incorrect recombination. Special care should be taken to avoid DNA crosslinking by UV-light, a blue-light gel analysis table was used for all experiments. For colony PCR, colonies were picked into 30 µl 0.1× Tris-EDTA and 1 µl was used in a 25 µl PCR reaction. DNA standards were included from Hyperladder (Bioline). All primers were HPLC purity (MetaBion, Martinsried, Germany) and primer sequences for the construction of Tubb3 are included in Table S1.

#### Yeast genomic retrieval

200 pmol of each complementary primer were combined in 1× NEB buffer 1, heated to 95 ^o^C for 5 minutes, and annealed slowly on at room temperature on benchtop. For BAC constructs, annealed primers were combined with 2 µg cut (*Bsr*GI, *Swa*I, or *Not*I cut; New England Biolabs) or uncut DNA (ImaGenes, *Uhrf1*, RPCIB731A06223Q; *Gfap*, RPCIB731E07463Q) and transformed into yeast with 1 µg linearized pRTVIR. For genomic DNA retrieval, annealed primers were combined with 100 µg pure digested genomic DNA prepared from E14TG2a (129/Ola) or JM8A1.N3 (C57BL/6) cells by phenol:chloroform extraction and transformed with 1 µg linearized pRTVIR. Colony PCR and plasmid rescue were carried out as above. Inclusion of the EGFP-promoter-neomycin cassette was carried out by PCR amplification with primers containing end sequences from pEGFP-N1 (Clontech) and selection was performed on 20 µg/mL kanamycin plates. Primer sequences are included in Table S1.

### Differentiation TauEGFPhyg Cells

Differentiation of mouse ES was carried out as described in [42]. Briefly, 5×10^5^ cells were plated on 6 well dishes in N2B27 neural differentiation medium. Media was changed every day for 8 days and cells were fixed and stained with anti-GFP (Aves Labs) and anti-Tubb3 (Abcam) to confirm co-localization.

### ES Cell Electroporation

Electroporation of E14TG2a (ATCC) and JM8A1.N3 [43] cells was carried out as described [4]. The exceptionally high gene targeting frequency is due to a well-established targeting pipeline in projects contributing to the International Knockout Mouse Consortium [3]. In brief, media was changed the night before electroporation, 2.5 µg of targeting vector was linearized and resuspended in 50 µl sterile PBS and electroporated in triplicate into 1×10^7^ ES cells (E14Tg2A.4 or JM8A1.N3) in a BTX electroporator (700V, 400Ω, 25 µF, pulse count at 1) and transferred to plates and distributed evenly. The next day, cells were examined for growth and changed to media containing selection antibiotic G418 125 µg/mL. 10 days following electroporation, cell colonies were picked, trypsinized and expanded. Genomic DNA preparations and long-range PCR were performed as described [4] to characterize correct recombination events. Primer sequences are included in Table S1.

## Supporting Information

Figure S1 **Multiple fragment targeting vector assembly of pRTVIR-Tubb3-TauEGFPhyg.** (**A**). Junction colony PCR on yeast clones demonstrating correctly recombined clones are shown with corresponding primers (Table S1) and expected sizes compared to DNA standards. (**B**). Restriction digests of correctly assembled vectors are shown along with indicated enzymes and expected sizes. (**C**). Targeting in E14 ES cells is demonstrated by long range PCR between two independent primers binding to the genomic region outside the targeting arms and an internal primer as shown together with expected sizes.
(TIF)

Figure S2 **Genomic retrieval and integration of EGFP-SV40-Neomycin cassette into pRTVIR vectors.** (**A**). Colony junction PCR. *Left side:* Restricted pRTVIR was transformed together with genomic DNA into competent yeast and colonies were evaluated for correctly recombined clones using junction colony PCR. *Right side:* Undigested vectors containing *Uhrf1* and *Gfap* genomic DNA were co-transformed with a PCR fragment containing EGFP-SV40-Neomycin into competent yeast and bacterial colonies were evaluated by junction colony PCR. (**B**). Restriction digests of correctly recombined vectors are shown along with indicated enzymes and expected sizes. (**C**). Targeting in ES cells is demonstrated by long range PCR between two independent primers binding on each genomic side (four total) outside the targeting arms and two respective internal primers as shown together with expected sizes. Order is LR5a, LR5b, LR3a, LR3b.
(TIF)

Figure S3 **EGFP fluorescence in** ***Uhrf1-*** **and** ***Gfap*** **-EGFP-SV40-Neomycin targeted ES cell lines.** Brightfield and epifluorescent images of ES cells and partially differentiated neuronal monolayers (ND) for *Uhrf1* and *Gfap* targeted lines are shown. No fluorescence is observed in the *Gfap* image, due to the likely absence of astroglial cells.
(TIF)

Figure S4 **Multiple sequence alignment of** ***Gfap*** **.** Sequencing of JM8 and E14-derived yeast vectors revealed two polymorphic regions in the 3′ UTR (brackets) of an alternatively spliced transcript of *Gfap*, terminating at exon 8a.
(TIF)

Table S1 **Primers used in vector construction and genotyping.**
(DOC)

## References

[1] Capecchi MR (1989) Altering the genome by homologous recombination. Science 244: 1288–1292. 2660260 10.1126/science.2660260

[2] Eppig JT, Blake JA, Bult CJ, Kadin JA, Richardson JE, et al (2012) The Mouse Genome Database (MGD): comprehensive resource for genetics and genomics of the laboratory mouse. Nucleic Acids Research 40: D881–D886. 22075990 10.1093/nar/gkr974 PMC3245042

[3] Bradley A, Anastassiadis K, Ayadi A, Battey JF, Bell C, et al (2012) The mammalian gene function resource: the International Knockout Mouse Consortium. Mamm Genome 23: 580–586. 22968824 10.1007/s00335-012-9422-2 PMC3463800

[4] Skarnes WC, Rosen B, West AP, Koutsourakis M, Bushell W, et al (2011) A conditional knockout resource for the genome-wide study of mouse gene function. Nature 474: 337–342. 21677750 10.1038/nature10163 PMC3572410

[5] Zou J, Mali P, Huang X, Dowey SN, Cheng L (2011) Site-specific gene correction of a point mutation in human iPS cells derived from an adult patient with sickle cell disease. Blood 118: 4599–4608. 21881051 10.1182/blood-2011-02-335554 PMC3208277

[6] Howden SE, Gore A, Li Z, Fung HL, Nisler BS, et al (2011) Genetic correction and analysis of induced pluripotent stem cells from a patient with gyrate atrophy. Proc Natl Acad Sci U S A 108: 6537–6542. 21464322 10.1073/pnas.1103388108 PMC3080993

[7] Cheng LT, Nagata S, Hirano K, Yamaguchi S, Horie S, et al (2012) Cure of ADPKD by selection for spontaneous genetic repair events in Pkd1-mutated iPS cells. PLoS One 7: e32018. 22347511 10.1371/journal.pone.0032018 PMC3276537

[8] Sebastiano V, Maeder ML, Angstman JF, Haddad B, Khayter C, et al (2011) In situ genetic correction of the sickle cell anemia mutation in human induced pluripotent stem cells using engineered zinc finger nucleases. Stem Cells 29: 1717–1726. 21898685 10.1002/stem.718 PMC3285772

[9] Soldner F, Laganiere J, Cheng AW, Hockemeyer D, Gao Q, et al (2011) Generation of isogenic pluripotent stem cells differing exclusively at two early onset Parkinson point mutations. Cell 146: 318–331. 21757228 10.1016/j.cell.2011.06.019 PMC3155290

[10] Yusa K, Rashid ST, Strick-Marchand H, Varela I, Liu PQ, et al (2011) Targeted gene correction of alpha1-antitrypsin deficiency in induced pluripotent stem cells. Nature 478: 391–394. 21993621 10.1038/nature10424 PMC3198846

[11] Liu Y, Rao M (2011) Gene targeting in human pluripotent stem cells. Methods Mol Biol 767: 355–367. 21822888 10.1007/978-1-61779-201-4_26

[12] Meyer M, Ortiz O, Hrabe de Angelis M, Wurst W, Kuhn R (2012) Modeling disease mutations by gene targeting in one-cell mouse embryos. Proc Natl Acad Sci U S A 109: 9354–9359. 22660928 10.1073/pnas.1121203109 PMC3386067

[13] Carbery ID, Ji D, Harrington A, Brown V, Weinstein EJ, et al (2010) Targeted genome modification in mice using zinc-finger nucleases. Genetics 186: 451–459. 20628038 10.1534/genetics.110.117002 PMC2954478

[14] Meyer M, de Angelis MH, Wurst W, Kuhn R (2010) Gene targeting by homologous recombination in mouse zygotes mediated by zinc-finger nucleases. Proc Natl Acad Sci U S A 107: 15022–15026. 20686113 10.1073/pnas.1009424107 PMC2930558

[15] Cong L, Ran FA, Cox D, Lin S, Barretto R, et al (2013) Multiplex Genome Engineering Using CRISPR/Cas Systems. Science 339: 819–823. 23287718 10.1126/science.1231143 PMC3795411

[16] Vasquez KM, Marburger K, Intody Z, Wilson JH (2001) Manipulating the mammalian genome by homologous recombination. Proc Natl Acad Sci U S A 98: 8403–8410. 11459982 10.1073/pnas.111009698 PMC37450

[17] Deng C, Capecchi MR (1992) Reexamination of gene targeting frequency as a function of the extent of homology between the targeting vector and the target locus. Mol Cell Biol 12: 3365–3371. 1321331 10.1128/mcb.12.8.3365-3371.1992 PMC364584

[18] te Riele H, Maandag ER, Berns A (1992) Highly efficient gene targeting in embryonic stem cells through homologous recombination with isogenic DNA constructs. Proc Natl Acad Sci U S A 89: 5128–5132. 1594621 10.1073/pnas.89.11.5128 PMC49242

[19] Sarov M, Schneider S, Pozniakovski A, Roguev A, Ernst S, et al (2006) A recombineering pipeline for functional genomics applied to Caenorhabditis elegans. Nat Methods 3: 839–844. 16990816 10.1038/nmeth933

[20] Nedelkova M, Maresca M, Fu J, Rostovskaya M, Chenna R, et al (2011) Targeted isolation of cloned genomic regions by recombineering for haplotype phasing and isogenic targeting. Nucleic Acids Res 39: e137. 21852329 10.1093/nar/gkr668 PMC3203589

[21] Suzuki K, Imai Y, Yamashita I, Fukui S (1983) In vivo ligation of linear DNA molecules to circular forms in the yeast Saccharomyces cerevisiae. J Bacteriol 155: 747–754. 6307979 10.1128/jb.155.2.747-754.1983 PMC217746

[22] Gibson DG, Benders GA, Axelrod KC, Zaveri J, Algire MA, et al (2008) One-step assembly in yeast of 25 overlapping DNA fragments to form a complete synthetic Mycoplasma genitalium genome. Proc Natl Acad Sci U S A 105: 20404–20409. 19073939 10.1073/pnas.0811011106 PMC2600582

[23] Storck T, Kruth U, Kolhekar R, Sprengel R, Seeburg PH (1996) Rapid construction in yeast of complex targeting vectors for gene manipulation in the mouse. Nucleic Acids Res 24: 4594–4596. 8948655 10.1093/nar/24.22.4594 PMC146279

[24] Gibson DG (2009) Synthesis of DNA fragments in yeast by one-step assembly of overlapping oligonucleotides. Nucleic Acids Res 37: 6984–6990. 19745056 10.1093/nar/gkp687 PMC2777417

[25] Bhargava J, Shashikant CS, Carr JL, Juan H, Bentley KL, et al (1999) Direct Cloning of Genomic DNA by Recombinogenic Targeting Method Using a Yeast-Bacterial Shuttle Vector, pClasper. Genomics 62: 285–288. 10610723 10.1006/geno.1999.6000

[26] Raymond CK, Sims EH, Olson MV (2002) Linker-mediated recombinational subcloning of large DNA fragments using yeast. Genome Res 12: 190–197. 11779844 10.1101/gr.205201 PMC155262

[27] Gillum AM, Tsay EY, Kirsch DR (1984) Isolation of the Candida albicans gene for orotidine-5′-phosphate decarboxylase by complementation of S. cerevisiae ura3 and E. coli pyrF mutations. Mol Gen Genet 198: 179–182. 6394964 10.1007/BF00328721

[28] Miller BG, Hassell AM, Wolfenden R, Milburn MV, Short SA (2000) Anatomy of a proficient enzyme: the structure of orotidine 5′-monophosphate decarboxylase in the presence and absence of a potential transition state analog. Proc Natl Acad Sci U S A 97: 2011–2016. 10681417 10.1073/pnas.030409797 PMC15745

[29] Iraqui I, Vissers S, Bernard F, de Craene JO, Boles E, et al (1999) Amino acid signaling in Saccharomyces cerevisiae: a permease-like sensor of external amino acids and F-Box protein Grr1p are required for transcriptional induction of the AGP1 gene, which encodes a broad-specificity amino acid permease. Mol Cell Biol 19: 989–1001. 9891035 10.1128/mcb.19.2.989 PMC116030

[30] Donnelly ML, Hughes LE, Luke G, Mendoza H, ten Dam E, et al (2001) The ‘cleavage’ activities of foot-and-mouth disease virus 2A site-directed mutants and naturally occurring ‘2A-like’ sequences. J Gen Virol 82: 1027–1041. 11297677 10.1099/0022-1317-82-5-1027

[31] Oldenburg KR, Vo KT, Michaelis S, Paddon C (1997) Recombination-mediated PCR-directed plasmid construction in vivo in yeast. Nucleic Acids Res 25: 451–452. 9016579 10.1093/nar/25.2.451 PMC146432

[32] Sikorski RS, Hieter P (1989) A system of shuttle vectors and yeast host strains designed for efficient manipulation of DNA in Saccharomyces cerevisiae. Genetics 122: 19–27. 2659436 10.1093/genetics/122.1.19 PMC1203683

[33] DiGtoP. Available: http://www.digtop.de/.

[34] van Deursen J, Wieringa B (1992) Targeting of the creatine kinase M gene in embryonic stem cells using isogenic and nonisogenic vectors. Nucleic Acids Res 20: 3815–3820. 1508665 10.1093/nar/20.15.3815 PMC334053

[35] Christianson TW, Sikorski RS, Dante M, Shero JH, Hieter P (1992) Multifunctional yeast high-copy-number shuttle vectors. Gene 110: 119–122. 1544568 10.1016/0378-1119(92)90454-w

[36] Goldstein AL, Pan X, McCusker JH (1999) Heterologous URA3MX cassettes for gene replacement in Saccharomyces cerevisiae. Yeast 15: 507–511. 10234788 10.1002/(SICI)1097-0061(199904)15:6<507::AID-YEA369>3.0.CO;2-P

[37] Falcon AA, Rios N, Aris JP (2005) 2-micron circle plasmids do not reduce yeast life span. FEMS Microbiol Lett 250: 245–251. 16085372 10.1016/j.femsle.2005.07.018 PMC3586270

[38] Gietz RD, Woods RA (2002) Transformation of yeast by lithium acetate/single-stranded carrier DNA/polyethylene glycol method. Methods Enzymol 350: 87–96. 12073338 10.1016/s0076-6879(02)50957-5

[39] Grant SG, Jessee J, Bloom FR, Hanahan D (1990) Differential plasmid rescue from transgenic mouse DNAs into Escherichia coli methylation-restriction mutants. Proc Natl Acad Sci U S A 87: 4645–4649. 2162051 10.1073/pnas.87.12.4645 PMC54173

[40] Rodriguez I, Feinstein P, Mombaerts P (1999) Variable patterns of axonal projections of sensory neurons in the mouse vomeronasal system. Cell 97: 199–208. 10219241 10.1016/s0092-8674(00)80730-8

[41] Szymczak AL, Workman CJ, Wang Y, Vignali KM, Dilioglou S, et al (2004) Correction of multi-gene deficiency in vivo using a single ‘self-cleaving’ 2A peptide-based retroviral vector. Nat Biotechnol 22: 589–594. 15064769 10.1038/nbt957

[42] Ying QL, Stavridis M, Griffiths D, Li M, Smith A (2003) Conversion of embryonic stem cells into neuroectodermal precursors in adherent monoculture. Nat Biotechnol 21: 183–186. 12524553 10.1038/nbt780

[43] Pettitt SJ, Liang Q, Rairdan XY, Moran JL, Prosser HM, et al (2009) Agouti C57BL/6N embryonic stem cells for mouse genetic resources. Nat Methods 6: 493–495. 19525957 10.1038/nmeth.1342 PMC3555078

[44] Kelley LA, Sternberg MJ (2009) Protein structure prediction on the Web: a case study using the Phyre server. Nat Protoc 4: 363–371. 19247286 10.1038/nprot.2009.2

