# Supplementary figures and images for: Direct Cloning of Isogenic Murine DNA in Yeast and Relevance of Isogenicity for Targeting in Embryonic Stem Cells
Source: PLoS One. 2013 Sep 13;8(9):e74207. doi: 10.1371/journal.pone.0074207 (PMC3772885; doi:10.1371/journal.pone.0074207)

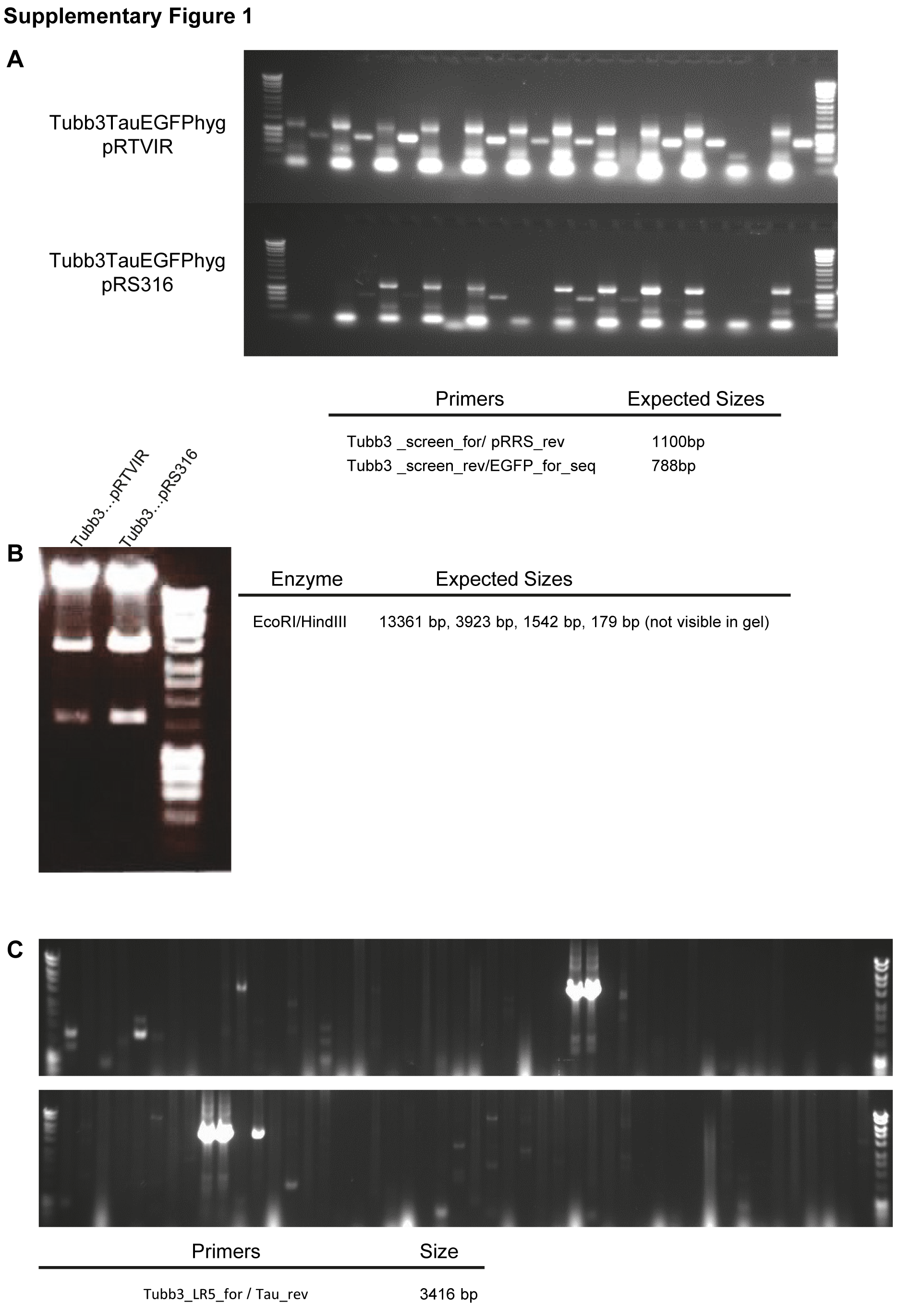

Supplement: Figure S1 — Multiple fragment targeting vector assembly of pRTVIR-Tubb3-TauEGFPhyg. (A). Junction colony PCR on yeast clones demonstrating correctly recombined clones are shown with corresponding primers (Table S1) and expected sizes compared to DNA standards. (B). Restriction digests of correctly assembled vectors are shown along with indicated enzymes and expected sizes. (C). Targeting in E14 ES cells is demonstrated by long range PCR between two independent primers binding to the genomic region outside the targeting arms and an internal primer as shown together with expected sizes. (TIF) [file pone.0074207.s001.tif]

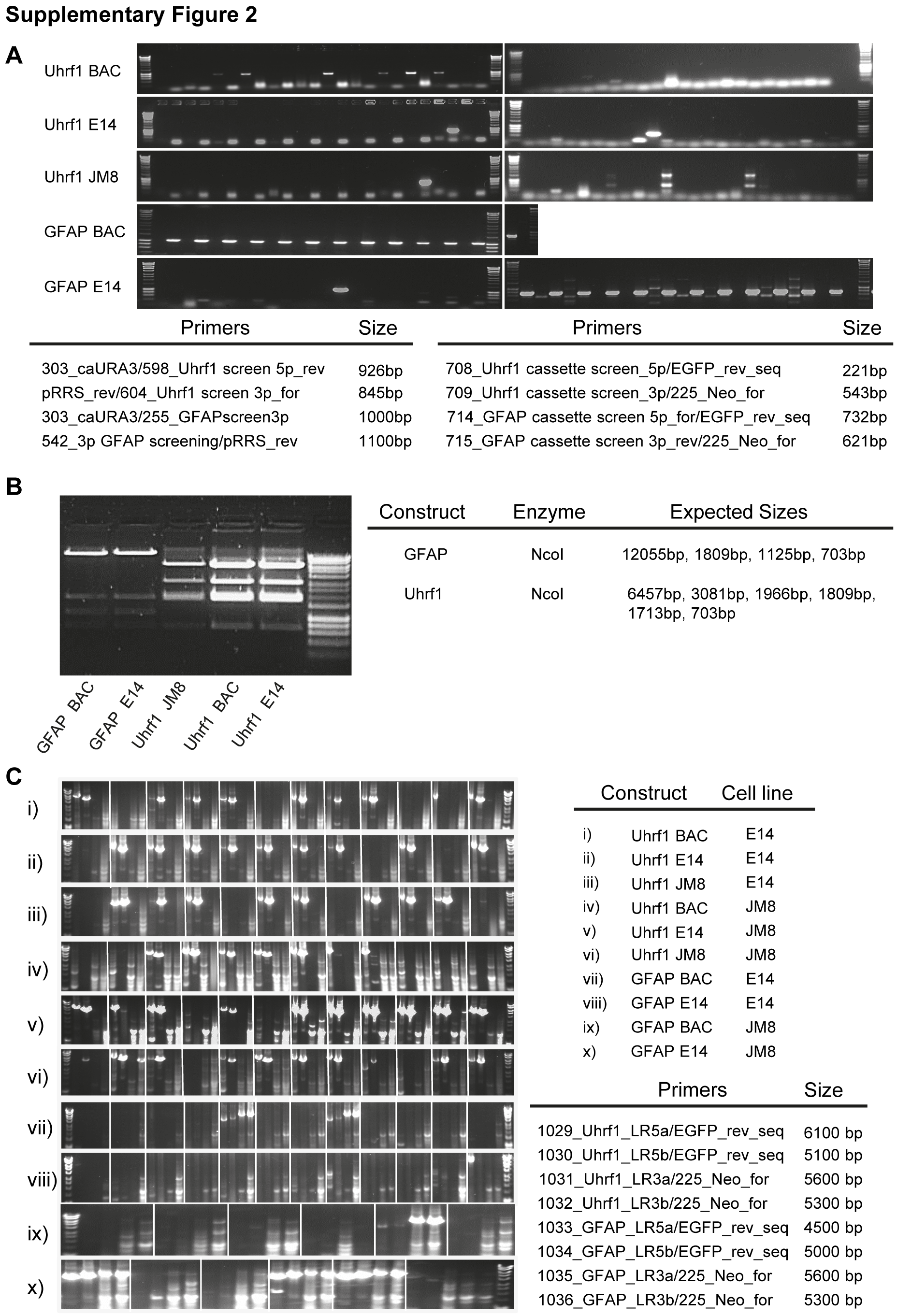

Supplement: Figure S2 — Genomic retrieval and integration of EGFP-SV40-Neomycin cassette into pRTVIR vectors. (A). Colony junction PCR. Left side: Restricted pRTVIR was transformed together with genomic DNA into competent yeast and colonies were evaluated for correctly recombined clones using junction colony PCR. Right side: Undigested vectors containing Uhrf1 and Gfap genomic DNA were co-transformed with a PCR fragment containing EGFP-SV40-Neomycin into competent yeast and bacterial colonies were evaluated by junction colony PCR. (B). Restriction digests of correctly recombined vectors are shown along with indicated enzymes and expected sizes. (C). Targeting in ES cells is demonstrated by long range PCR between two independent primers binding on each genomic side (four total) outside the targeting arms and two respective internal primers as shown together with expected sizes. Order is LR5a, LR5b, LR3a, LR3b. (TIF) [file pone.0074207.s002.tif]

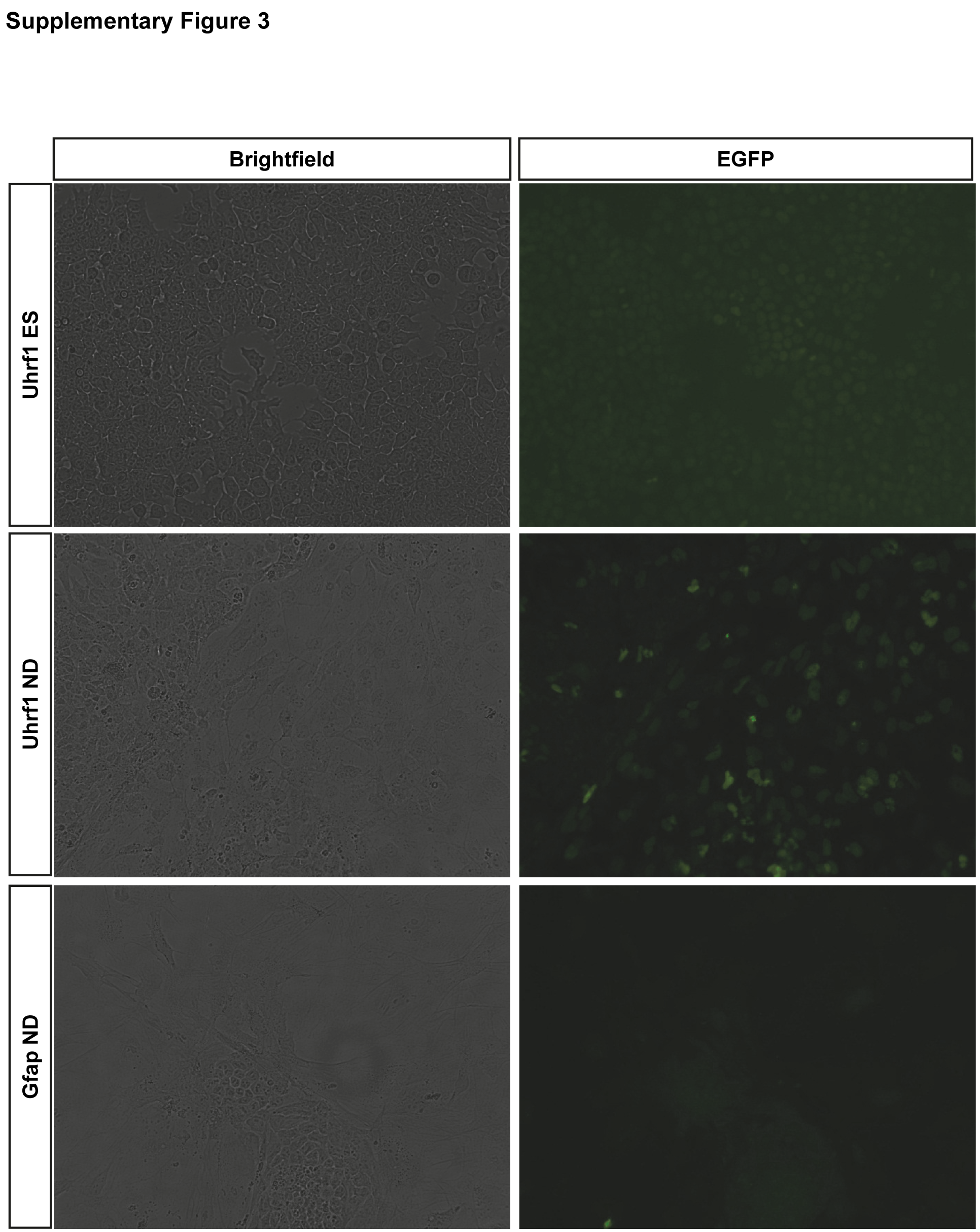

Supplement: Figure S3 — EGFP fluorescence in Uhrf1- and Gfap -EGFP-SV40-Neomycin targeted ES cell lines. Brightfield and epifluorescent images of ES cells and partially differentiated neuronal monolayers (ND) for Uhrf1 and Gfap targeted lines are shown. No fluorescence is observed in the Gfap image, due to the likely absence of astroglial cells. (TIF) [file pone.0074207.s003.tif]

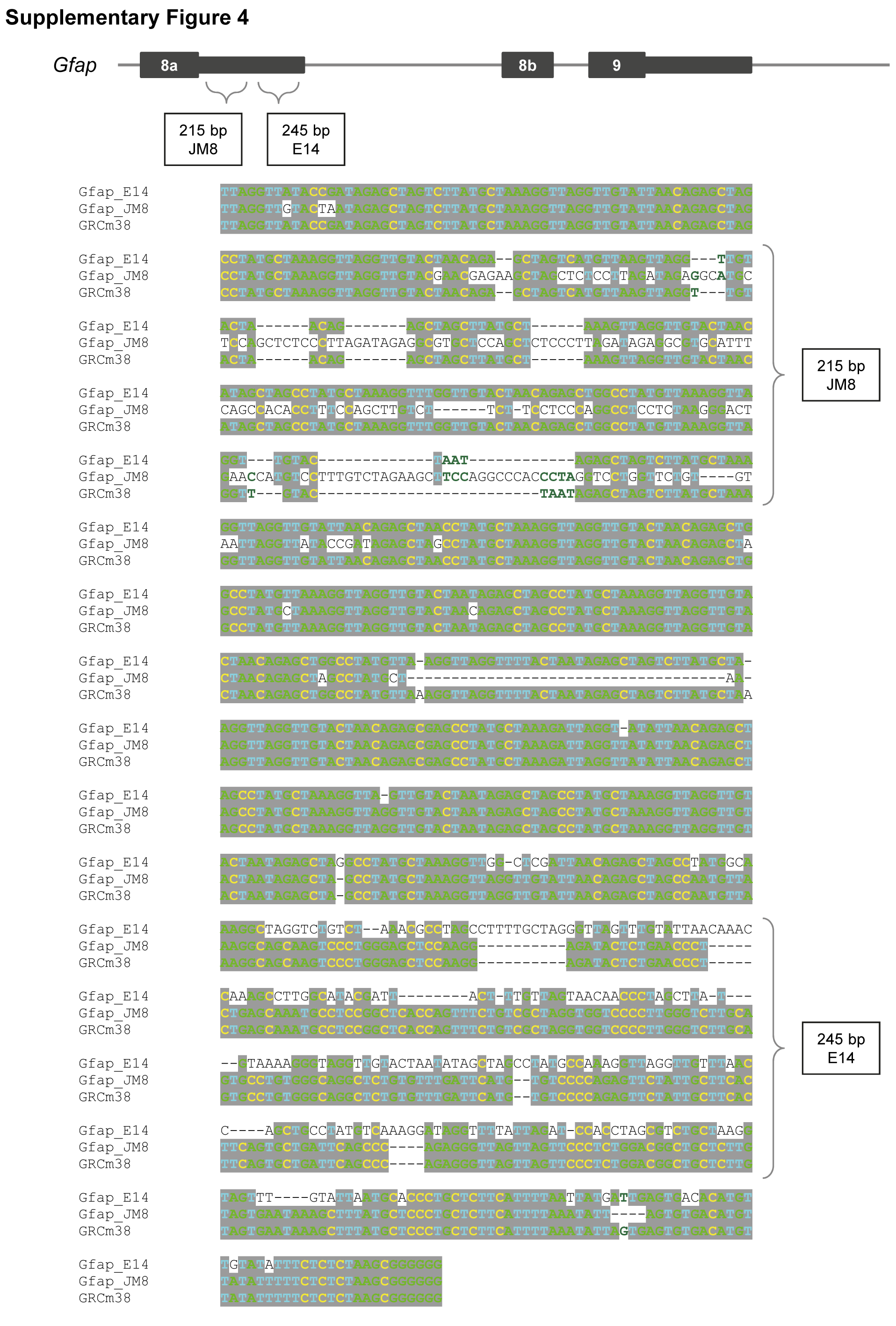

Supplement: Figure S4 — Multiple sequence alignment of Gfap . Sequencing of JM8 and E14-derived yeast vectors revealed two polymorphic regions in the 3′ UTR (brackets) of an alternatively spliced transcript of Gfap, terminating at exon 8a. (TIF) [file pone.0074207.s004.tif]
