# Supplementary material for: Direct Cloning of Isogenic Murine DNA in Yeast and Relevance of Isogenicity for Targeting in Embryonic Stem Cells
Source: PLoS One. 2013 Sep 13;8(9):e74207. doi: 10.1371/journal.pone.0074207 (PMC3772885; doi:10.1371/journal.pone.0074207)
Supplement: Table S1 — Primers used in vector construction and genotyping. (DOC) [file pone.0074207.s005.doc]

| **Table S1. Primers used in vector construction and genotyping** | |
| --- | --- |
| **pRTVIR** |  |
| A | AGCTGTTTCCTGTGTGAAATTG |
| B | GCTAGAAAGTATAGGAACTTC |
| C | CGCTTTTGGTTTTCAAAAGCGCTCTGAAGTTCCTATACTTTCTAGCATTTAGCCCATACATCC |
| D | GGAATTGTGAGCGGATAACAATTTCACACAGGAAACAGCTGATATCTTATAATTGGCCAGTCTTTTTC |
| E | GAAAAAGACTGGCtAAgctTAAGATATCAGCTGTTTCC |
| F | GCTTATTTGAAAAAGtaaGcttAATTATAAGATATCAG |
| G | TTGGAATGCTTATTTGtAAgcttCTGGCCAATTATAAG |
|  |  |
| **TauEGFPhyg** |  |
| Tubb3_fragment_1_for | tgaattgtaatacgactcactatagggcgaattggagctccattgatgattgactaggccgtcct |
| Tubb3_fragment_1_rev | cctccacgtcgccgcaagtcagcaggctgccgcggccctccttgggcccctgggcttccgattcct |
| Tubb3_fragment_2_for | gtcgagataacttcgtatagcatacattatacgaagttattgaagttgctcgcagctggggtgt |
| Tubb3_fragment_2_rev | cggaattaaccctcactaaagggaacaaaagctgggtacccctcgtcctgggactacatcacagt |
| Tubb3_fragment_3_for | gagggccgcggcagcctgctgacttgcggcgacgtggaggagaaccccggccccatggctgaccctcgccaggagtt |
| Tubb3_fragment_3_rev | ccacatcgccacaggttagcagcgagcctcggccctccttgtacagctcgtccatgccga |
| Tubb3_fragment_4_for | gaggctcgctgctaacctgtggcgatgtggaagagaatccaggcccgatgaaaaagcctgaactcaccgcgacgt |
| Tubb3_fragment_4_rev | agaaacataacttcgtataatgtatgctatacgaagttatttattcctttgccctcggacgagt |
| Tubb3_fragment_5_for | gaataaataacttcgtatagcatacattatacgaagttatgtttctagcttggctgcaggtc |
| Tubb3_fragment_5_rev | tactgccccgccctcggggatggatccacagacctgcggctacaagaaagctgggtctagatatctcga |
| Colony PCR |  |
| Hyg_for | cagaagcgcggccgtctgga |
| Neo_rev | tgcgtgcaatccatcttgttc |
| Tubb3 _screen_for | ttgagaggccacagagcagttagctg |
| pRRS_rev | cattaatgcagctggcacgac |
| Tubb3 _screen_rev  EGFP_for_seq | tatgatctagagtcgcggccgctttattcctttgccctcggacgagt  AGAAGCGCGATCACATGGTCCTGCT |
| Genotyping Primers |  |
| Tubb3_LR5_for | GCTTGATTGAGGCTACTTGATAATTGATACAGT |
| Tubb3_LR5pB | CAGGCAGGAGACTCAATGATACATCCTTAGCA |
| Tau_rev | tgctggccacacgagcttgagtcacat |
|  |  |
| **Uhrf1** |  |
| Genomic retrieval and colony PCR |  |
| 600_Uhrf1 SwaI_NotI retrieval pCA775_5p | aggtaaaaggtatagagatgctggttggaatgcttatttgaaaaagactggctaacagacagtagcctctggcaggccctggcctatgctaggga |
| 601_Uhrf1 SwaI_NotI retrieval pCA775_5pRC | tccctagcataggccagggcctgccagaggctactgtctgttagccagtctttttcaaataagcattccaaccagcatctctataccttttacct |
| 602_Uhrf1 SwaI_NotI retrieval pCA775_3p | ggcggcgctcagcagcttccaggcccctttggggggccgcagctgtttcctgtgtgaaattgttatccgctcacaattcc |
| 603_Uhrf1 SwaI_NotI retrieval pCA775_3pRC | ggaattgtgagcggataacaatttcacacaggaaacagctgcggccccccaaaggggcctggaagctgctgagcgccgcc |
| 303_caURA3 | tagaactgttgatgaagttgttagcactgga |
| 598_Uhrf1 screen 5p_rev | tctctgctcaaagccacaggc |
| 604_Uhrf1 screen 3p_for 2nd primer | agacagcgattgacattcagtcg |
| pRRS_rev | cattaatgcagctggcacgac |
| 656_UHRF1 Swa Not pRS316 | tgaattgtaatacgactcactatagggcgaattggagctccagacagtagcctctggcaggccctggcctatgctaggga |
| 657_UHRF1 Swa Not pRS316 | tccctagcataggccagggcctgccagaggctactgtctggagctccaattcgccctatagtgagtcgtattacaattca |
| 658_UHRF1 Swa Not pRS316 | ggcggcgctcagcagcttccaggcccctttggggggccgcggtacccagcttttgttccctttagtgagggttaattccg |
| 659_UHRF1 Swa Not pRS316 | cggaattaaccctcactaaagggaacaaaagctgggtaccgcggccccccaaaggggcctggaagctgctgagcgccgcc |
| Cassette Inclusion |  |
| 794_UHRF1 EGFP NEO | ccttgcagaccattctcaaccagctcttccctggctatggcagcggccggggatcaggcagcggttctgggtcaggatcaggctccatggtgagcaagggcgaggagct |
| 795_UHRF1 EGFP NEO | acacactatgaagttggaaccatgagccctctgcctgggtctcagcatcagaagaactcgtcaagaaggcgataga |
| 708_Uhrf1 cassette screen_5p | Tcgctttgagctggaccacagc |
| EGFP_rev_seq | cacgctgaacttgtggccgtttac |
| 709_Uhrf1 cassette screen_3p | Aactcccctctgcaatcatgtcagtg |
| 225_Neo_for | tcccgattcgcagcgcatcgcct |
| Long range genomic PCR |  |
| 1029_Uhrf1_LR5a | GAGGCTCTGTCTCATCAGGCACT |
| 1030_Uhrf1_LR5b | CCTATTTCCATAGACAGTGGTGTCTAACACT |
| EGFP_rev_seq | cacgctgaacttgtggccgtttac |
| 1031_Uhrf1_LR3a | CAGGGACAGATGGCTACCTAGTGCT |
| 1032_Uhrf1_LR3b | GATATGCACAGGTACATGGAGCACCT |
| 225_Neo_for | tcccgattcgcagcgcatcgcct |
|  |  |
| **GFAP** |  |
| Genomic retrieval and colony PCR |  |
| 488_Gfap_hom_BsrGI_pCA775_5p | aggtaaaaggtatagagatgctggttggaatgcttatttgaaaaagactggctaagtacagactttctccaacctccagatccgaggtcagtata |
| 489_Gfap_hom_BsrGI_pCA775_5pRC | tatactgacctcggatctggaggttggagaaagtctgtacttagccagtctttttcaaataagcattccaaccagcatctctataccttttacct |
| 490_Gfap_hom_BsrGI_pCA775_3p | tagagtgagtcagagcttgctccgagaaaagtaaaatagtagctgtttcctgtgtgaaattgttatccgctcacaattcc |
| 491_Gfap_hom_BsrGI_pCA775_3pRC | ggaattgtgagcggataacaatttcacacaggaaacagctactattttacttttctcggagcaagctctgactcactcta |
| 275_GFAP pRS202 | gtgaattgtaatacgactcactatagggcgaattggagctcaactgatgccaggtctgcaggctatgaccaagctctgcc |
| 276_GFAP pRS202 | ggcagagcttggtcatagcctgcagacctggcatcagttgagctccaattcgccctatagtgagtcgtattacaattcac |
| 277_GFAP pRS202 | gagaggagggcctgagaaaaatagcaagccaacctgaactccagcttttgttccctttagtgagggttaattccgagctt |
| 278_GFAP pRS202 | aagctcggaattaaccctcactaaagggaacaaaagctggagttcaggttggcttgctatttttctcaggccctcctctc |
| 255_GFAPscreen3p | tacaacctaacttaacatgactagctctgt |
| 303_caURA3 | tagaactgttgatgaagttgttagcactgga |
| 542_3p GFAP screening primer F | ggtacccacatggtggacatacatacat |
| pRRS_rev | cattaatgcagctggcacgac |
| Cassette Inclusion |  |
| 970_GFAP EGFP NEO | ccctgcaggtcattaaggactcgaagcaggagcacaaggacgtggtgatgggatcaggcagcggttctgggtcaggatcaggctccatggtgagcaagggcgaggagct |
| 971_GFAP EGFP NEO | ctttgggccctcacactgcatggcaagggccaccaggtgggcacacctcatcagaagaactcgtcaagaaggcgataga |
| 714_GFAP cassette screen 5p_for | agctctaactgggacagttggg |
| EGFP_rev_seq | cacgctgaacttgtggccgtttac |
| 715_GFAP cassette screen 3p_rev | atcctgccaagtgctgagaacc |
| 225_Neo_for | tcccgattcgcagcgcatcgcct |
| Long Range genomic PCR |  |
| 1033_GFAP_LR5a | CTGCAGGAGTACCAGGATCTACTCA |
| 1034_GFAP_LR5b | CAAGCCAAGCACGAAGCTAACGACT |
| EGFP_rev_seq | cacgctgaacttgtggccgtttac |
| 1035_GFAP_LR3a | CTGAAGACTTAAGCTCTGCTGACATGCA |
| 1036_GFAP_LR3b | CCGTCAACAAGTGTAGCCAAGTAGCT |
| 225_Neo_for | tcccgattcgcagcgcatcgcct |
|  |  |
|  |  |
